# Development, implementation and evaluation of a smartphone application aimed to reduce sedentary time and increase physical activity among Indian sedentary office workers – findings from SMART-STEP trial

**DOI:** 10.1186/s12889-025-23049-9

**Published:** 2025-05-16

**Authors:** Baskaran Chandrasekaran, Ashokan Arumugam, Arto J. Pesola, Poornima Panduranga Kundapur, Chythra R. Rao

**Affiliations:** 1 Department of Exercise and Sports Sciences, Manipal College of Health Professions, Manipal Academy of Higher Education, Manipal, Karnataka 576104 India; 2 Department of Physiotherapy, College of Health Sciences, University of Sharjah, P.O. Box: 27272, Sharjah, United Arab Emirates; 3 Neuromusculoskeletal Rehabilitation Research Group, RIMHS – Research Institute of Medical and Health Sciences, University of Sharjah, P.O. Box: 27272, Sharjah, United Arab Emirates; 4 Sustainable Engineering Asset Management Research Group, RISE - Research Institute of Sciences and Engineering, University of Sharjah, P.O. Box: 27272, Sharjah, United Arab Emirates; 5 Adjunct Faculty, Department of Physiotherapy, Manipal College of Health Professions, Manipal Academy of Higher Education, Manipal, Karnataka India; 6 Active Life Lab, South-Eastern Finland University of Applied Sciences, 50100 Mikkeli, Finland; 7 Department of Data Science and Computer Applications, Manipal Institute of Technology, Manipal Academy of Higher Education, Manipal, Karnataka 576104 India; 8 Department of Community Medicine, Kasturba Medical College, Manipal, Manipal Academy of Higher Education, Manipal, Karnataka 576104 India

**Keywords:** Smartphone application, Sedentary time, Acceptability, Sustainable industry, Safe work, Productivity

## Abstract

**Background:**

Excessive sedentary time (ST) is linked to an increased risk of cardiometabolic diseases. Although various behavioral interventions have emerged to reduce ST, the potential of smartphone (SmPh)-based interventions remains relatively unexplored in workplace settings. This study aimed to explore the development, implementation and acceptability of a SmPh application among Indian desk-based office workers.

**Methods:**

One hundred thirty-six office workers were randomised to one of three interventions for six months: (1) SmPh-based ST and physical activity (PA) reminders (SMART); (2) traditional education (TRADE) and (3) usual work group (CONT). A threshold of 70% adherence (equivalent to responding to at least 580 out of 828 assigned prompts) was used to classify participants as ‘compliant’. Of 44 SMART group participants who were assigned to 24 weeks of intervention, nine participants were purposefully selected based on compliance, Moreover, they were interviewed for potential barriers associated with the SmPh application using semi-deductive approach.

**Results:**

The SMART STEP application was developed over eight months, during which three versions were created and pilot tested. The cost of application development was reasonable (≈ $1,860). Of 44 participants who received SmPh application-based reminders, 37 completed the two follow-ups at 3rd and 6th month. Mean prompt engagement rates, defined as ‘the percentage of prompts participants actively responded to’, during the 1st, 2nd, 3rd, 4th, 5th, and 6th months were 77% (*n* = 107), 59% (*n* = 82), 54% (*n* = 74), 45% (*n* = 63), 43% (*n* = 59), and 31% (*n* = 43), respectively. Barriers such as workload, lack of movement sensing, and insufficient organizational and peer support were key factors contributing to the decline in long-term engagement among office workers.

**Conclusion:**

The SMART-STEP application appears to be an affordable and promising solution for reducing ST and promoting PA among office workers in low-resource settings. However, enhancements such as embedding movement sensing technology, organizational policies and peer education are needed to improve long-term usability and acceptability.

**Trial registration:**

Clinical Trial Registry of India (CTRI/2020/03/024138) registered on 20/03/2020.

**Supplementary Information:**

The online version contains supplementary material available at 10.1186/s12889-025-23049-9.

## Background

Prolonged sedentary time (ST), defined as any waking time characterized by low energy expenditure of less than 1.5 metabolic equivalents while in a sitting or a reclined posture [1], and insufficient physical activity (PA), defined as engaging in less than 150 min of moderate-to-vigorous PA per week, MVPA [2] are now recognized as independent risk factors for a myriad of chronic diseases, including diabetes, coronary artery disease, hypertension, and cancer [3]. Hence public health guidelines underscore the importance of reducing ST and increasing MVPA for reducing chronic disease risk [2]. Although MVPA [defined as activity exceeding three metabolic equivalents (METs)] is known for its health benefits, most individuals in modern desk-based jobs struggle to meet the recommended 150 min of MVPA in a week [4]. Consequently, average MVPA levels remains low, a situation further exacerbated by higher ST, which collectively elevates the risk of chronic diseases among sedentary office workers. Recent accelerometer-based studies have confirmed that desk-based office workers spend approximately 73% of their working hours sedentary [5–8]. During their total waking hours, they remain sedentary for about 59% to 66% of the time [5–8]. Interrupting prolonged sedentary bouts with light-intensity PA (LIPA), defined as activity that expends between 1.6 and 2.9 metabolic equivalents, is viewed as equally beneficial to MVPA in countering cardiometabolic disease risk [9]. While MVPA offers significant benefits for aerobic fitness and cardiometabolic health, maintaining such intensity levels in everyday settings can be challenging, often resulting in low adherence to prescribed exercise regimens [10]. In contrast, LIPA is more feasible and less daunting, particularly for older adults and individuals with chronic conditions, as it demands less energy expenditure and is easier to integrate into daily routines [11]. Moreover, interventions promoting LIPA tend to be more enjoyable, leading to higher adherence rates [12].

Though numerous behavioral interventions have been developed to reduce ST and PA at workplaces, Chu et al., 2016 has divided the interventions into three categories: (1) individual level behavioral counselling – goal setting, action planning [13]; (2) environmental level—installing active workstation; (3) multicomponent interventions combining the above two interventions [14]. Environmental and multicomponent interventions incur costs, require expertise, and necessitate higher organizational policy-level changes. To break a habitual activity like sitting, which requires no conscious effort, interventions involving LIPA should be simple, easily delivered and adapted into everyday lives [15, 16]. Smartphones (SmPh), being ubiquitous, is proposed to be an intriguing intervention for an effective behavior change at the individual level [17]. Though evidence on SmPh driven physical activities was remained unconvinced five years back [18], the findings of recent feasibility and field based trials confirm that SmPh is promising in reducing ST and increasing PA among end-users [19].

Mobile phone adoption continues to rise globally, with an estimated 5.6 billion unique mobile users as of 2023, surpassing the number of people with access to electricity or personal vehicles in many regions [20]. This widespread penetration, particularly in low- and middle-income countries like India, makes smartphones a promising tool for scalable digital health interventions. Observational studies claimed that more than 27 million urban Indian adults own android based SmPhs accounting for 9% of the total SmPh users across the globe [21]. With significant advanced in the geo-navigational systems, search engines, cameras and social media usage, the SmPhs influence the habitual behavior of Indians including movement behaviors however the large potential remains untapped.

According to the Global Burden of Disease Study 2019, non-communicable diseases (NCDs) such as cancer, diabetes, obesity, hypertension, stroke, and coronary artery disease are on the rise in Indian communities, contributing significantly to early mortality and disability-adjusted life years [22]. Physical inactivity, estimated at 41.4% in the Indian population based on the National NCD Monitoring Survey (2017–2018) [23], has now emerged as the third leading risk factor for NCDs, following tobacco use and unhealthy diet [22]. Large-scale community surveys also indicate that workplace PA constitutes a major source of total PA among rural populations, in contrast to urban residents [23]. Furthermore, India’s rapidly expanding digital market presents a unique opportunity to develop engaging and scalable PA interventions aimed at reducing physical inactivity and associated NCD risk in the population [24–26].

**Fig. 1:**
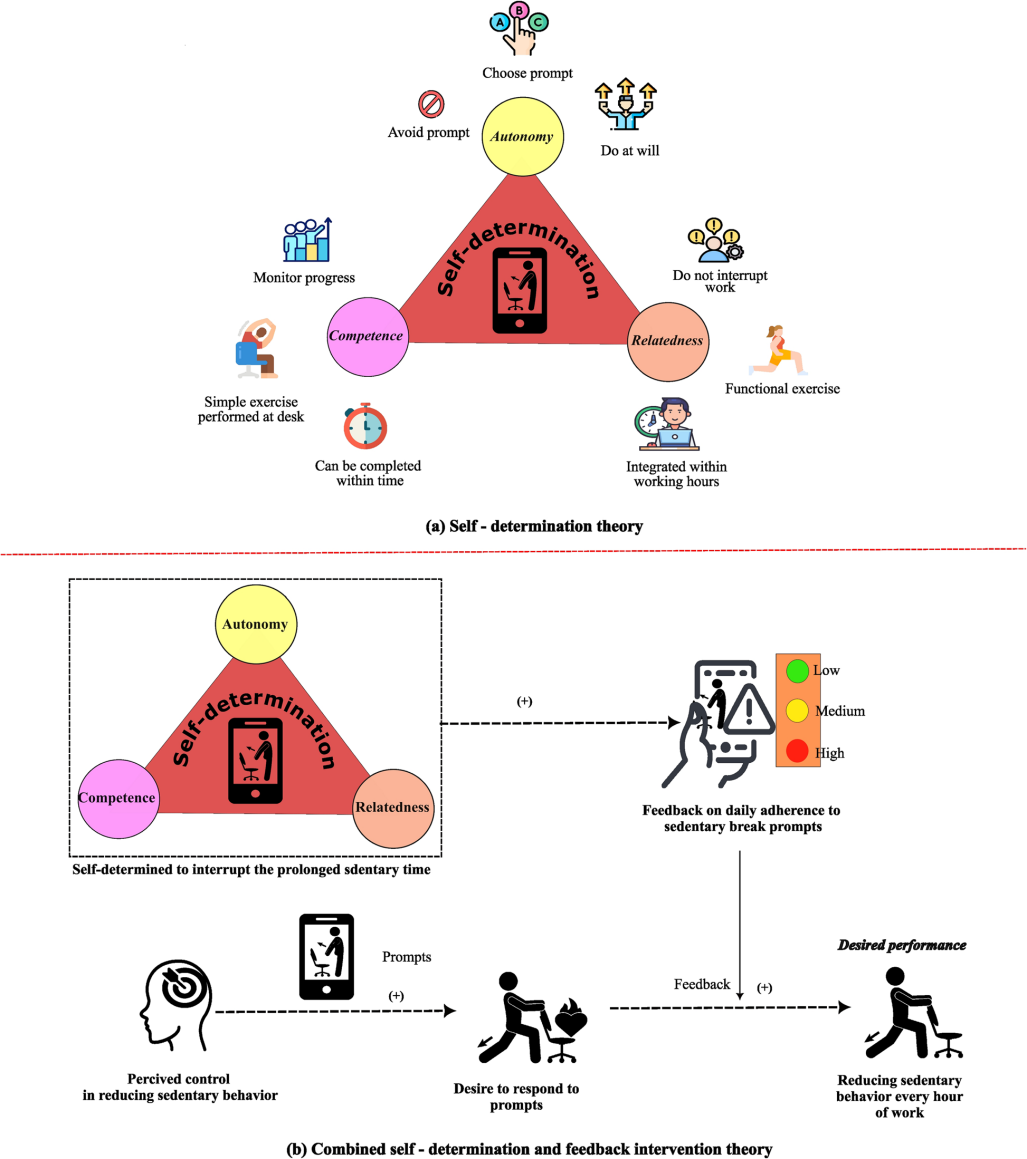
Theoretical basis of smartphone application development. Initial smartphone application development was based on self-determination model with the three constructs (autonomy, competence and relatedness). Later feedback intervention model was iterated as the feedback might improve adherence to the desired movement behaviors

SMART-STEP trial is a cluster randomized controlled trial from India that aimed to investigate the effectiveness of SmPh-based reduction in ST and increase in PA among office workers of sedentary workplaces in India [27, 28]. The idea behind the SmPh application development was to provide nudges or prompts to interrupt their ST and to perform PA every hour for two minutes during their 9–5 PM working hours which translates to 16 min of PA and usually recommended by previous experimental studies [29, 30]. The SmPh application was developed as per the tenets of the theoretical principles laid by self-determination theory and feedback intervention theory considering the previous studies that administered digital technologies to reduce ST at workplace [31]. ‘Self-determination theory’ undermines human motivation and personality that suggest the individuals can be self-determined to perform a behavior when three innate psychological needs are fulfilled: autonomy, competence and relatedness [32]. The SmPh application aimed to improve the office worker’s autonomy (can choose or avoid the prompt and can be done at will based on the situation and task priority), competence [simple exercises that can be performed at the desk without expert supervision, completed within a specified time, and monitored through color-coded daily adherence levels indicating progress in reducing ST and increasing PA) and improve relatedness (should not interrupt daily work flow, exercises that are simple and functional and integrated within working hours so that it does not interrupt non-working hours). Figure 1a depicts the development of the SmPh application based on Self-Determination Theory, highlighting its core components: autonomy, competence, and relatedness. According to ‘feedback intervention model’ [33], once the office worker desired and self-determined to perform the desired behavior (reduce ST and increase PA at workplace), the application should provide feedback on the present adherence to prompts [three color coded icons – low (green), moderate (yellow) and high (red)], so that the office workers could modify or maintain their current behavior levels (ST and PA).

Figure 1b illustrates the SmPh application components, which were developed based on the Feedback Intervention Model. We chose to ground the intervention in individual-level behavior change theories specifically, Self-Determination Theory [32] and Feedback Intervention Theory [33], because this was the first digital intervention of its kind targeting PA and ST among Indian office workers in real-world settings. Broader socio-ecological or policy-level theories (e.g., Social Cognitive Theory, Social Ecological Models) were not employed as the intervention was not implemented at the organizational or community level, and structural changes (e.g., policy adaptations, environmental modifications) might not be possible as our intervention is foundational paving the way for future theory expansion. Table 1 summarizes the operationalization of Self-determination theory and Feedback-Intervention model constructs within the SMART-STEP application.

**Table 1:** Operationalization of Self-determination theory and Feedback-Intervention model constructs within the SMART-STEP application

Theoretical model and constructs		Features embedded in the SMART-Step
Self-determination Theory	Autonomy	• Prompt flexibility • Can skip the prompts based on task priority
	Competence	• Achievable desk-based exercises • Visual progress tracking
	Relatedness	• Embedding prompts in ways that did not disrupt the office environment
Feedback-Intervention Model	Feedback	• Feedback causing shifting attention from work to performing movement break
	Task learning	• Displaying simple callisthenic exercises every time the participant login to learn the exercise
	Task motivation	• Hourly reminders of exercises displayed at the home page of the Smartphone screen
	Meta-task	• Color coded (engaged > 4 prompts, green—good; engaged < 2 prompts, red – bad) provides motivation to perform more exercise breaks

The customized SmPh-based application which was developed for SMART-STEP trial, is described here to aid future developers and public health experts to develop cost effective SmPh-based PA promotion for sedentary office workers. Furthermore, the acceptability and usability of the customized SmPh-based application was explored in the participants who completed 24 weeks of a three arm cluster randomised controlled trial (SMART-STEP), the details can be found in the previous publications [28, 34].

The primary study aimed to evaluate the effectiveness of a technology-based intervention (SMART) for reducing ST/increasing PA behaviors compared to traditional workplace counselling (TRADE) and a control group (CONT), using accelerometer-measured ST/PA levels and cardiometabolic risk markers. The protocol, primary outcomes, and process evaluation of this cluster randomized controlled trial have been previously published [5, 27, 28, 34, 35]. In contrast, the present paper focuses on detailing the development process of the SmPh application used in the SMART intervention, which delivered ST/PA reminders during work hours, and examines its usability and acceptability among Indian desk-based office workers.

## Methods

The manuscript conformed to the reporting guidelines based on “mobile health (mHealth) evidence reporting and assessment (mERA) checklist” [36]. The checklist is reported as supplementary file S1. The study on development and the acceptability of SmPh-based intervention among office workers was conducted between March 2021 and November 2023. The Institutional Ethical Committee (IEC:749/2019) approved the study, and the study was prospectively registered in the Clinical Trial Registry of India (CTRI/2020/03/024138). The research was conducted as per the ethical principles laid by the tenets of Helsinki [37]. SMART-STEP followed three phases that aimed: (1) to develop SmPh application to provide ST/PA break reminders during work hours; (2) to assess the effectiveness of the intervention through the cluster randomised controlled trial; (3) to explore the acceptability and usability of the SmPh application among the Indian office workers [27]. The findings of the effectiveness of the SmPh application on objectively measured ST/PA levels and acceptability can be accessed elsewhere [28, 34].

### Developing the exercise regime for smartphone prompts

A comprehensive review of contemporary literature on exercises suitable for inclusion in the SMART-STEP trial was conducted by the primary author (BC) between September 2018 and February 2019 during the trial’s planning phase. The exercises were adapted based on the previous literature which administered workplace ST/PA interventions and recommended dose for potential health benefits [38, 39].

The decision to provide exercise breaks every hour was informed by a combination of prior empirical evidence and practical considerations relevant to sedentary workplace settings [19, 40, 41]. Previous studies have demonstrated that interrupting prolonged sitting every 30–60 min with short bouts of PA, even as brief as 2–3 min, can yield meaningful benefits in cardiometabolic and musculoskeletal health markers [19, 42]. Additionally, hourly breaks were considered a feasible option for integration into standard office workflows without disrupting productivity, as supported by feasibility studies conducted in occupational settings [19, 40]. The exercise modality alternating between simple strengthening and stretching activities was chosen to enhance adherence, reduce monotony, and address common musculoskeletal complaints among Indian desk-based workers [43]. While the core framework was adapted from Sjøgaard et al. [25], it was supplemented by contemporary guidelines and intervention designs described in recent workplace PA literature [19, 40, 42].

Each hourly break prompt delivered by the SMART-STEP application consisted of two exercises: one strengthening and one stretching exercise. These were performed during working hours (9:00 AM – 5:00 PM), totaling approximately 16 min of activity per day. The strengthening exercises were designed to be completed in about one minute. Each involved performing a single set of 15 repetitions, with each repetition lasting 3–4 s. For exercises requiring unilateral performance (e.g., leg curls, hip raises), two sets were done with 7–8 repetitions per side. The stretching exercises were also designed to last one minute, comprising two repetitions of a 30-s static stretch per muscle group, one repetition per side. All exercises could be performed in a standing position at the workstation without requiring additional equipment or supervision. The detailed breakdown of each exercise, including frequency, intensity, and volume, is provided in Table 2. The supplementary file S2 shows the pictorial representation of both body-supported exercises and stretching exercises that were embedded in the SmPh application.

**Table 2:** Exercises that were adapted for break reminders in both interventions (SMART & TRADE)

Exercise^a^	Frequency	Intensity	Duration	Volume	Sides
*Strengthening exercises* (displayed each repetition 3–4 s and the exercises were repeated 15–20 times in the SmPh screen)					
Leg curl	Every one hour	Perceived exertion of 8–10 of Borg RPE scale	One minute	3- 4 s/rep; 15 repetitions/minute	Both legs (8 rep each leg)
Squats					Not applicable
Desk push-ups					Not applicable
Lunges					Both legs (8 rep each leg)
Dips					Not applicable
Single hip raises					Both legs (8 rep each leg)
*Stretching exercises* (displayed each repetition 30 s and the exercises were repeated 2 times in the SmPh screen)					
Triceps	Every one hour	Pain free stretch until the physiological limit	One minute	30 s per stretch, two stretches	Both arms (one stretch per arm)
Forearm flexors					
Latissimus dorsi					
Pectoralis					
Quadriceps femoris					Both legs (one stretch per side)
Hamstrings					

*Rep* Repetition, *RPE* Rating of perceived exertion, *SMART* Group that received smartphone driven sitting time reminders and pedometer driven step-based interventions, *TRADE* Group that received education manual on strategies to improve physical activity and reduce sitting time

^a^The exercises were adapted from Sjøgaard et al., 2014 [32]

### Design and development of smartphone application (SMART-STEP)

**Fig. 2:**
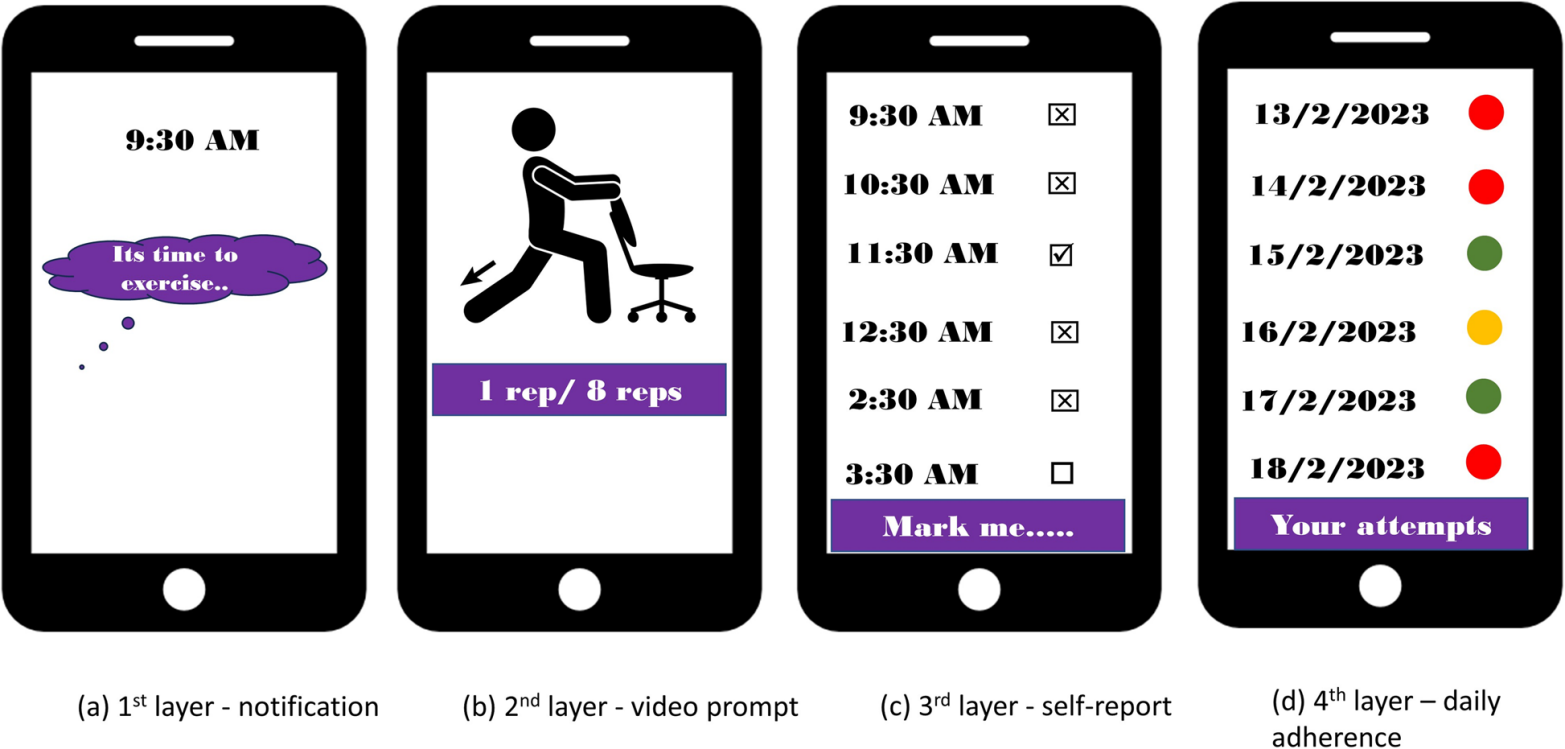
Navigation layers designed after consensus met within the interdisciplinary development team

The SMART-STEP application was designed by an interdisciplinary research team comprising of public health experts, community intervention behavioral scientists and software developers. The application was purposefully designed for SmPh running on Android operating systems. Observational studies have demonstrated that Android users account for more than 90% of SmPh users in India [44, 45]. As Android based SmPhs’ are common, can be customized at low-cost, feature-richer and cost-effective, we designed SmPh application for office workers with Android SmPhs. The application development process consisted of four processes using pragmatic reiterative processes: 1) deciding the specifications of the application with the developer, 2) creating the design using wireframes and mock-ups regarding notification prompts, 3) selection of appropriate platform (Android, version 5.0 and above), developing XML layouts and SQ-lite for user log interactions, and 4) testing the prototype at alpha and beta levels. The application was developed on the JAVA platform and was saved as.apk files. The SmPh application was developed to ensure compatibility with all Android processors (v5.0 and later) and to operate seamlessly in both offline and online modes. The application was designed so that end-users can monitor their compliance daily through color coding: green (attempted more than four out of six daily prompts), orange (attempted two to four daily prompts) and red (less than two daily prompts). Figure 2 demonstrates the hypothesized navigation layers for the SMART-STEP application.

### Development of webhosting

**Fig. 3:**
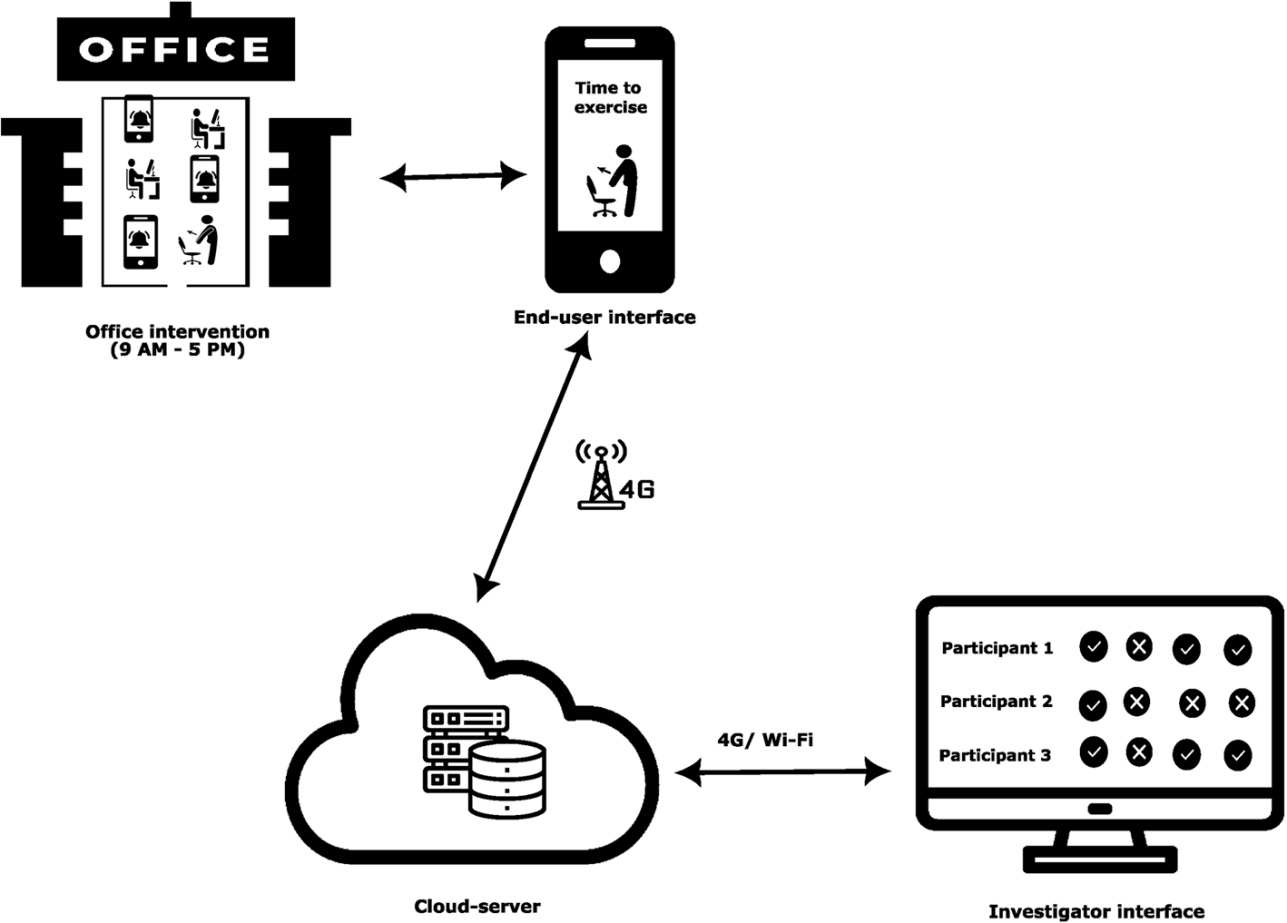
Final ideation of Smartphone application development, navigation panels and its synchronization with the customized central server for the participant adherence monitoring

Further a web portal (SMART-STEP) was developed for synchronization of the data from the individual end-user SmPhs’. Figure 3 illustrates the different navigation panels adapted for the SmPh application and the synchronization with the web version for the primary investigator to monitor. The web version showed the compliance (intention to perform the exercise) of the end-user to the hourly prompts and the data was stored on the secure server of the developer with appropriate subscription for the investigators. Any bug fixation and the issues arising during alpha testing were resolved with reiteration and appropriate versions were released.

### User testing

Alpha testing was carried out within the developing team for nearly two months prior to the SMART-STEP trial to assess the acceptability of user interface, any technical complaints including but not limiting battery drain, timing of the prompts, prompt loudness, video loops for exact time, hanging up of screen, network issues, compliance visualization and web synchronization. Members of the development team (two software developers, one community intervention behavioral scientist, two public health experts) were carrying the application installed SmPhs’ for several days together to understand the timing, pattern of prompts and technical concerns regarding video reminders, quality of the video and prompt beep sounds. Two alpha versions were developed after fixing bugs and improving video quality while retaining the resolution. Further beta testing in three typical office workers revealed video streaming difficulties and annoying prompt sounds. The bugs were again fixed, alarms were silenced and only vibrating notifications to the main screen were allowed. Three beta versions were developed during this reiteration period. The prompts synchronization to the web server discussed earlier was also tested at alpha level.

### Implementation

After the development and successful alpha and beta level testing, SmPh-based intervention was implemented on a large scale as a cluster randomised controlled trial which is explained elsewhere [27, 28] and summarized below.

#### Participants

Desk-based office workers of fifteen administrative clusters of a large multifaceted university were contacted through appropriate advertisements in the university noticeboards and exchange users. To be eligible for participants should be of age 30–50 years, 8 h of full-time office work with computer interaction, spent atleast 60% of their workday in sitting and physically inactive (not meeting the weekly dose of > 150 min/week in MVPA). Exclusion criteria included the presence of chronic diseases, acute trauma, or pregnancy that could limit participation in physical activity. Individuals working night shifts or planning to change jobs during the trial period were also excluded. Furthermore, participants randomized to the SmPh-based reminders group were required to own an Android SmPh running version 5.0 or higher to facilitate the installation of the intervention application.

#### Cluster randomised controlled trial (SMART-STEP trial)

The study details and the baseline characteristics of the participants included in the SMART-STEP trial, have been reported earlier [5, 27, 28]. The administrative offices of 15 institutional clusters of an university were contacted for necessary approval to recruit their desk-based office workers satisfying the above eligible criteria. The clusters, which varied in size, were randomly assigned to one of the three intervention groups in ascending order based on the number of workers per cluster, resulting in approximately equal participant distribution across the SMART, TRADE, and CONT groups:

1. *SMART group:* Participants received the SMART-STEP application installed on their personal Android SmPh (devices were not provided), which delivered hourly reminders during office hours to reduce ST. Each reminder included a 2-min activity consisting of one stretching and one strengthening exercise (see Supplementary File S2). Upon responding to a notification, users’ application interactions were synced with a secure external server. Additionally, this group participated in a pedometer-based step challenge of 10,000 steps per day target, recording their daily step count in a logbook submitted every two weeks.
2. *TRADE group:* Participants received a workplace education manual outlining the risks of prolonged sitting, benefits of reducing ST, and practical strategies for increasing PA during and outside of work hours. They also maintained a daily log to record their walking time.
3. CONT group: Participants in this group continued their usual work routine for six months without engaging in any new physical activity intervention.

The design and details of the protocol can be accessed elsewhere [27]. At baseline, 1 st, 3rd and 6 th month follow up, body composition, cardiometabolic & musculoskeletal health measures along with work productivity were measured. Furthermore, seven-day ST/PA levels were measured by hip worn accelerometer (Actigraph wGT3X-BT) at baseline, 3rd and 6 th month, while biochemistry parameters were measured at baseline and at 6 th month. At the end of 6 months, a sub-group of participants who completed interventions (SMART, TRADE) were contacted for a qualitative interview about the barriers and enablers faced during the implementation of interventions at workplaces.

### Evaluation of the interventions

Usability was assessed using the application usage data at periodic intervals, especially week wise of 24 weeks. As the office workers push the touch screen for seeing the notification in their mobile phone, this was recorded as the ‘intention to PA or reduce ST,’ synchronized to the external server based on the network of the client. The ‘intention’ or ‘usage’ data was downloaded from the password protected server every weekend for all the SMART group participants.

To assess compliance, we defined two key operational terms. “Non-attempted prompts” referred to daily ST/PA notifications delivered by the application that were not interacted with by the participant (i.e., the prompt was neither tapped nor acknowledged). “Non-compliant participants” were those whose overall engagement rate across the six-month intervention period fell below the predefined threshold of 70% (equivalent to responding to fewer than 580 out of 828 assigned prompts) as determined by application log data. This threshold was adapted from prior exercise science literature [46, 47].

Of ninety office workers (SMART *n* = 44, TRADE, *n* = 46) completed six months of SMART and TRADE interventions, 18 office workers (SMART *n* = 9, TRADE, *n* = 9) were purposefully selected based on compliance and were invited to participate in the study. Included participants should articulate their views on the interventions, addressing their experiences and challenges. The details of the process evaluation and the qualitative findings can be accessed in our previous published literature [34]. For the qualitative evaluation of the SmPh application implemented which is the objective of the present paper, only the data pertained to the SMART groups were presented in the paper. Specifically, nine office workers were selected, with five categorized as “compliant” (engaged more than 70% (580/828) of the prompts in six months) and four as ‘non-compliant’ (used the application irregularly or discontinued not meeting the threshold). 70% exercise attendance cutoff was adopted based on thresholds reported in previous physical activity literature [46, 47]. Compliance was determined based on application usage logs synced to the server, which recorded the frequency and duration of prompt interactions. This sampling approach ensured inclusion of diverse user experiences.

Semi-structured interviews were conducted to explore potential barriers to application engagement. An interview guide was developed with signaling questions focusing on usability, convenience, and other contextual barriers related to the smartphone-based intervention. The final set of questions was collaboratively developed and approved by the three co-authors (CRR, AJP, and AA). Table 3 shows the questions asked during the interview, especially for the SmPh application users in SMART group. The original interview guide is provided as a supplementary file S2 in the recent publication [34].

**Table 3:** Probe questions used to assess the acceptability of the SMART-STEP smartphone application

Questions asked during the interview^a^	Signaling questions
Can the application be used in the current form?	Easy navigation, convenient in use
Do you have any problem with the phones while using?	Battery, crash, data/server problems
In what way can we improve the application that can motivate you?	Games, any sensors integrated
What are the barriers you faced with the intervention other than phones itself?^b^	Individual (task, laziness), interpersonal (colleague support) and organizational (task, load, policies)

^a^The questions were uniformly asked to all participants in the SMART group, who were the only group invited for this qualitative evaluation

^b^the last question explored contextual and behavioral barriers beyond technical issues to understand broader factors affecting application integration into users’ routines, which are critical for assessing real-world acceptability

Participants were interviewed in private areas within the vicinity of their workplace. All interviews were conducted by a trained researcher (BC) involved in the study. Participants were informed about the interviewer’s role in the research team at the beginning of the interview. All interviews were audio-recorded, transcribed verbatim, and analyzed using a deductive approach to categorize responses into constructs and sub-constructs based on the socio-ecological framework [34]. Data saturation was considered achieved when no new themes or insights emerged [48]. Qualitative data analysis was performed using Taguette, a web-based free and open-source coding tool and thematic analysis was undermined [34]. The identified themes were organized around three key levels influencing adherence to the SmPh-based sedentary time and physical activity (ST/PA) intervention: individual, interpersonal, and organizational, based on the socio-ecological model.

The following results section shows the findings: (i) the development process of the SmPh application, (ii) its implementation as a large-scale institutional trial, and (iii) the process evaluation findings from six months of intervention delivery.

## Results

### Smartphone application development

**Fig. 4:**
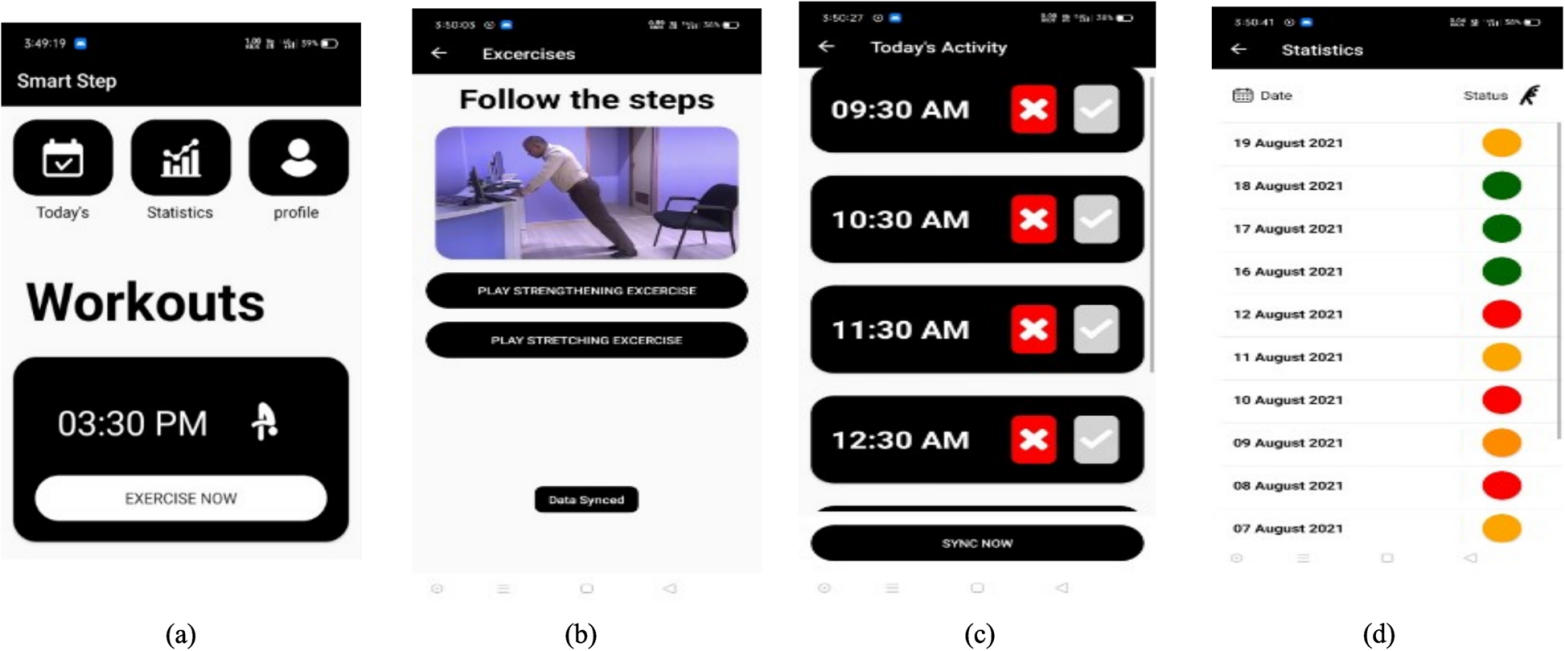
End user interface navigation panels of SMART-STEP application. Other than the welcome and login panel, the push button for the time (**a**), video cue (**b**), daily exercise goal setting (**c**) and the compliance panel () are displayed

**Fig. 5:**
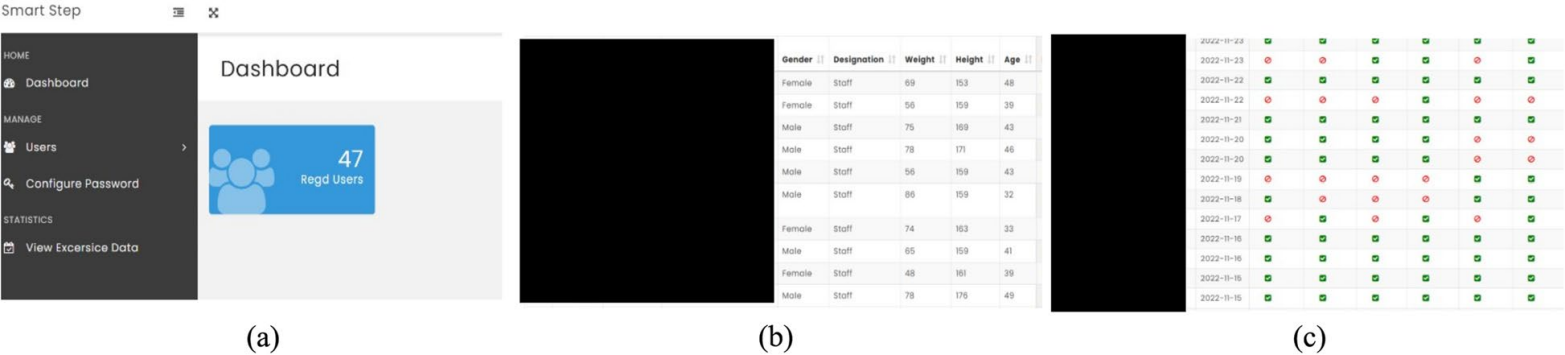
Web server hosting. The three navigation panels were (**a**) user registration page, (**b**) user profile and (**c**) adherence monitoring

The development and pilot testing of the SMART STEP application took eight months (July 2020 – February 2021). Three versions of the application were developed with the final beta version SMART-STEP (version 3.0). Alpha testing revealed bugs (video crash, synchronization) and issues (video loop over, weekend alarms, battery drain) which were resolved prior to beta testing. At the final alpha testing, the SmPh application prompts were working well with no error in the periodic cues and synchronization with the PHP servers. The final version of the SmPh application consisted of six navigation layers: welcome page, login page, notification override, push button page, exercise prompt page and compliance page (even when participants did not open but have done the exercise, were able to log their compliance panel later). Figure 4 depicts the end-user interface panels with the navigation layers. The data was stored offline and synchronized with the central server (http://smartstep.mohammedalfaz.com/) and server was protected by username and password shared only with investigators (Fig. 5). On first time login, the participants were given liberty of changing the password. Unlike the contemporary workplace PA promotion SmPh applications available [49], the designed application did not ask for any access to Wi-Fi, camera, contacts and the files and the data security in private server was overseen by the application development company (Buildreams, Karkala, Karnataka, India, https://buildreamsgroup.com/).

### Webhosting (PHP external server)

The developer designed four navigation panels for the administrator (primary author and co-authors access using username and password). The four navigation panels are as follows: (1) Dashboard providing overall summary of the log entry of the participants to view the prompts (compliance – daily, weekly and monthly displayed with the anonymous codes for participant identity); (2) Users: this page depicts the participants and demographic details; (3) Password: to reset the passwords for the clients who have formatted their phone at the middle of the trial; (4) View exercise data: this page consolidates the number of prompts in numeric form for the ease of data entry. The consolidated log can be downloaded as excel, csv and pdf by the authors. Figure 5 depicts the navigation panels; demographics of end-users and adherence monitor page. The cost incurred for SmPh application development (design, user testing) and private server (hosting, maintenance, report generation) for 16 months was translated to 1,860 USD which was considered a relatively low development cost compared to the typical operational budgets of medium- to large-sized organizations in India, particularly those in academic institutions and corporate information technology sectors, where digital infrastructure is already in place.

### SMART group participants characteristics in the randomised trial

**Fig. 6:**
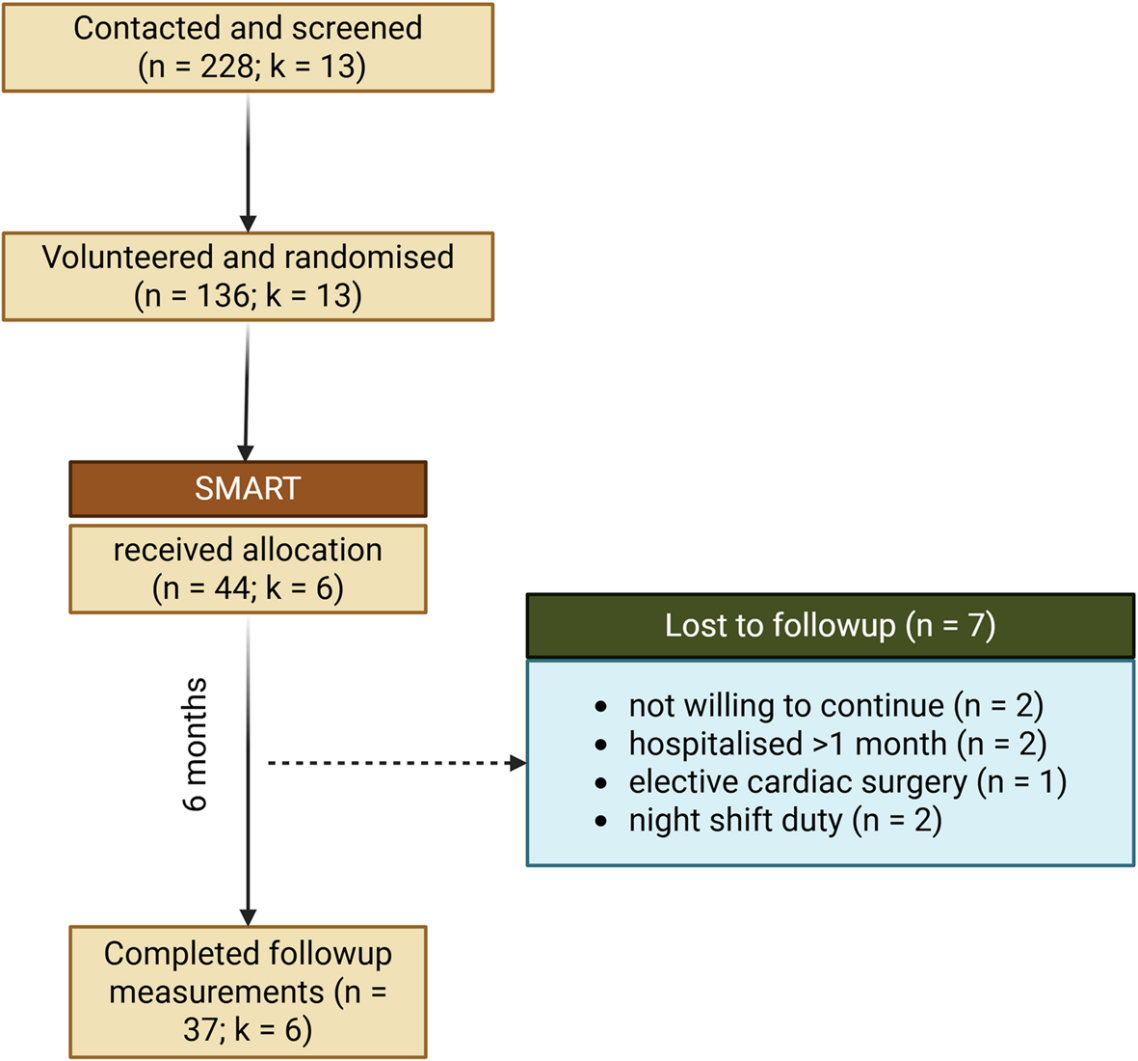
Screening and inclusion of the SMART group participants in SMART-STEP trial

Of the 136 participants from 13 clusters who were randomized to the SMART (*n* = 44), TRADE (*n* = 46), and CONT (*n* = 46) groups, 84% of those in the SMART group (*n* = 37) completed the six-month trial along with all four follow-up assessments (baseline, first, third, and sixth month). Figure 6 illustrates the screening process and inclusion of SMART group participants in the trial.

Participants in the SMART group exhibited the following baseline characteristics: median age [35 years (IQR: 30.75–35)], years of work experience [8 years (IQR: 2.75–8)], and daily calorie intake [2736 kcal (IQR: 2565.75–2837)]. The majority were female [*n* = 26, 59%], held postgraduate qualifications [*n* = 31, 71%], and were engaged in administrative roles involving customer interaction [*n* = 31, 71%]. There were no significant baseline differences in accelerometer-measured ST and PA levels between the groups. Participants in the SMART group recorded an average of 686.74 min/day of ST, 255.59 min/day of LIPA and 17.69 min/day of MVPA. Detailed baseline characteristics of the other two behavioral arms (TRADE and CONT) are available in our recently published paper [28].

### Usability of the application

**Fig. 7:**
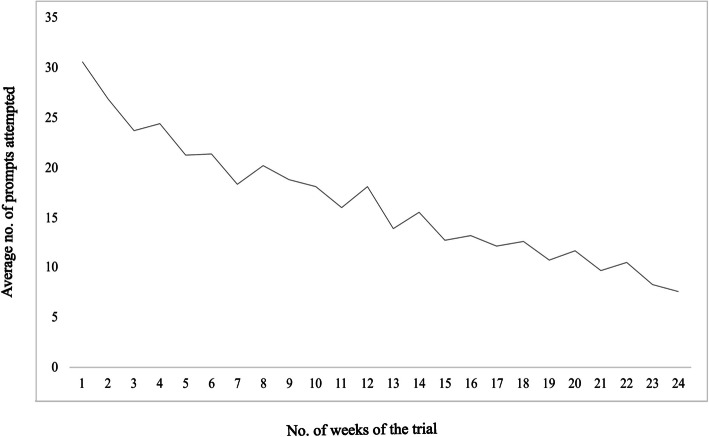
Average number of prompts attended by the SMART group. SMART refers to the group that received smartphone-based sitting time reminders and pedometer-driven, step-based interventions

In 24 weeks, a total of 828 SmPh prompts were delivered, with 138 prompts monthly excluding third Saturdays and Sundays (the University holidays). Mean attendance in response to prompts declined progressively over the six-month period: 77% (107 prompts) in the 1 st month, 59% (82 prompts) in the 2nd, 54% (74 prompts) in the 3rd, 45% (63 prompts) in the 4 th, 43% (59 prompts) in the 5 th, and 31% (43 prompts) in the 6 th month. Approximately half of the daily assigned breaks were not attempted. 34% of SMART group participants (*n* = 15/44) met the predefined threshold for sedentary break compliance. Weekly adherence to application prompts is illustrated in Fig. 7.

### Process evaluation of the application

Of the nine SMART participants who completed the interview, all the participants accepted the simplicity and the potential to influence the movement behaviors (ST and PA) however, few concerns regarding the applications were (1) simple design of the application; (2) perceived workload; (3) colleagues’ vicinity; (4) organizational policies.

#### Simple and easy design

All the participants (*n* = 9) agreed to the simplicity of the design as it provided a goal setting.

*“Smartphone reminders…. Not monotonous…… different exercise not repeating on the same day…. I liked the idea and did couple of times…..I missed few times” (P1, M, 46 years)*

The simplicity in design itself becomes a barrier for continuing to comply with the prompts. Some of the respondents (*n* = 3/9, 33%) felt that the prompts were rigid without sensing their movements.

*“I didn’t follow because it’s just an alarm…. that tells me what I must do” (P8, F, 42 years)*

*“Prompt is rigid… if I missed due to meetings and after resumed work, it does not sense……. When I want the prompt/clue was not there…. It should sense my sitting time…. Rather only rigid timing prompts….”. (P16, F, 44 years).*

#### Workload

Majority of the respondents (*n* = 7/9, 78%) prioritize their work instead of health. Few (*n* = 2/9, 22%) did not use their mobile phones during their work.

*“I immerse so much when I start work…. I ignore even calls…. These mobile notifications…...I ignore most……I want to complete my work assigned to me…. other things come next…” (P13, F, 37)*

As the intervention was carried out in administrative offices of healthcare and technology education sectors which are primarily consumer based rather than true desk-based office work, few respondents (*n* = 2/9, 22%) felt these interventions are better integrated into software and information technology offices.

#### Colleagues’ vicinity/private space

The usage of SmPh applications increased among participants who had private space for their administrative work while those who have shared workspaces (*n* = 6/9, 67%) felt shy to do exercises in front of their colleagues.

*“I am shy to do in front of others. It’s an open area you know…. When my cubicle is free…. Just me… I have done the exercises………” (P11, F, 34)*

Some felt they were not able to attend the prompts or exercises during official meetings or while meeting deadlines. Few (*n* = 2/9, 22%) expressed the need for social support to sustain the behaviors in addition to the video reminders.

*“there is no buddy or group in the app…. If people come together and share their success with colleagues…. Group dynamism…. Something like that… just a prompt…individual entity… It may not work…” (P16, F, 44).*

#### Organizational policies

Majority of the participants (*n* = 7/9, 78%) felt the rigidity in the work and scheduled break policies.

*“…. This is the work culture here………I knew few IT companies that allow their office workers to have recreational activities in between…. But here it is different and difficult…..” (P6, F, 39)*

Flexibility in standing during meetings and brief interruptions in work was not a social norm. Few (*n* = 2/9, 22%) perceived the need of work culture that allows for active meetings, awareness of sedentary work and health benefits with brief interruptions to sedentary behavior.

*“….. during meetings, it’s not a culture to even stand and I use to ignore the prompts…. I cannot stand and take notes, isn’t it?. Regular desks allow me only for sitting. It (organizational policies) is rigid here…..(P16, F, 44 years)*

Moreover, gender disparities in family roles emerged as a notable barrier to engage with the SmPh application–based ST/PA video reminders, as revealed in our qualitative interviews.

*“With my typing, listening to meeting tasks, I was already mentally exhausted. I reserve my physical energy for evening to prepare meals at night and looking after my younger daughter…. I need energy that I did not want to physically exhaust myself with exercises.” (P8, F, 42 years)*

## Discussion

The present study explored the development, usability and acceptability toward the customized SmPh application for SMART-STEP trial. The application was developed, and the activity was integrated into PHP server successfully. Although the application was developed based on contemporary research, the usage and acceptability of the SmPh prompts were found to be low, as several barriers emerged from our qualitative findings. Key barriers to optimize the SmPh based break reminders among Indian office workers included the lack of movement sensing, limited movement literacy among both employees and organizations, and the absence of a conducive environment and supportive social culture to promote PA and reduce ST in Indian workplace settings.

Experimental trials that have established favorable health benefits with SmPh-based PA interventions, have grounded strong behavioral theories such as feedback, goal setting, problem solving and social cognitive theories [33, 50]. A previous review has established that mobile health applications grounded in sound behavioral theory and incorporating techniques such as goal setting, feedback, motivational interviewing, and prompts for action are effective in promoting behavior change among office workers [49]. The SMART-STEP application was developed using individual-level behavior change theories specifically Self-Determination Theory and Feedback Intervention Theory, which supported autonomy, competence, and real-time feedback. However, despite this solid theoretical foundation, the intervention did not integrate broader social or environmental components, such as organizational support or policy-level facilitation. Prior literature indicates that the effectiveness and sustained usability of such interventions are enhanced when individual-level strategies are embedded within supportive organizational or ecological frameworks [51–54]. Thus, the relatively lower long-term engagement observed in our study may reflect the absence of these broader contextual supports. Future studies should consider integrating the SMART-STEP application within multilevel strategies to maximize effectiveness and usability.

Although participants appreciated the intuitive design, ease of navigation, and clarity of prompts, the static and non-personalized nature of the reminders contributed to perceptions of monotony and reduced novelty over time. This pattern of behavioral decay is well-documented in digital health literature [55, 56]. Incorporating context-aware features, such as movement sensing via embedded sensors, could allow for more tailored prompt delivery based on actual sedentary behavior rather than fixed schedules [56, 57]. Furthermore, incorporating strategies such as adding personalized feedback loops, gamification elements, and peer-support features (e.g., leaderboards, social challenges) may enhance user engagement and motivation [58, 59].

Significant reduction in SmPh logs were evident weekly. Majority of the prompts went unattended. This behavioral decay was commonly evident in workplace intervention studies due to several perceived barriers [60]. One potential reason is that our study involved sedentary workers of offices which intensively involved consumer interactions rather than computer interactions alone. Further the office workers involved in heterogenous administrative work (financial transaction, consumer help, designing) which might have influenced the perception of interrupting ST in workplaces. Nevertheless, social theories mapping, material reward, motivation and sensing ability using accelerometers or pressure sensors might have been the significant contributors to the poor engagement with the application [49, 55]. Further lack of awareness of activity literacy, lack of PA interruptions as a social culture in Indian workspaces were also speculated to be the barriers for the effective engagement with the digital interventions for workplace PA promotion [61]. Significant cultural variability, lack of awareness and perception about PA in the workplace was evident during the unstructured review where the participants expressed the opinion that occupational activity and leisure time PA could never be combined as the former may influence the routine work. Appropriate educational strategies to create awareness on need of such behavioral strategies at the workplace should be implemented before designing similar applications to increase motor behaviors in the workplaces of low-resource settings [13, 62].

While the development cost of the SMART-STEP application (USD 1,860 for 16 months) is relatively low for medium- to large-sized organizations, we acknowledge that smaller or resource-constrained workplaces particularly in public or non-profit sectors may face budgetary limitations. In such contexts, collaborative funding models, institutional cost-sharing, or integration with existing workplace wellness programs may help mitigate financial barriers and improve scalability [63]. Further, we recommend the SmPh-based reminders should be used as a part of multicomponent interventions in combination with other strategies such as workplace ST/PA counselling [13], installing active stations and workplace champions training may improve the adherence to workplace PA and ST interventions.

Although this study was conducted in the administrative settings of a university, the findings have important implications for broader non-academic and non-healthcare workspaces particularly in information technology (IT) and other corporate sectors where computer-based tasks dominate over consumer interaction, and employees often spend over 70% of their time in prolonged sitting bouts [64]. SmPh-based interventions may be especially relevant in corporate environments, where Android SmPh penetration is high and application based task management is already well integrated into daily workflows [65]. Additionally, digital tools grounded in individual behavior change models, such as our SMART-STEP application, offer scalable, low-cost solutions that require minimal logistical support in such contexts. Corporate offices may also allow greater flexibility in modifying physical environments (adoption of standing desks, flexible work hours, and active meetings) compared to academic institutions [66]. Integrating the SMART-STEP application with these supportive environmental and policy-level modifications could further enhance user engagement and adherence in corporate settings.

### Strength and limitations

To the best of our knowledge, the application development is first of its kind to reduce ST and increase PA at the same time among sedentary office workers in India. Further the SmPh application was developed at a low cost which is affordable and easy to implement among sedentary workspaces of low-resource settings. Our research may open avenues for new technology-based interventions to reduce ST and increase PA in densely populated country like India where the chronic disease risk is high. Besides strengths, few limitations of the study are (1) the application employed in the present study was grounded on individual theory (self-determination and feedback intervention) whereas recent studies which have employed social theories (social cognitive and social ecological models) reported significant success in reducing workplace ST and improving PA [67]; (2) the application lacked personalized messages or sensor based support which might have reduced the motivation in engaging in long term [56]; (3) As noted earlier, this study was conducted exclusively among university office workers. Therefore, the qualitative findings may not be generalizable to more heterogeneous populations, such as those in non-academic, non-university, or corporate settings. Future research should consider including diverse occupational groups to enhance the applicability and transferability of the results [28]; (4) Additionally, the qualitative interviews were conducted with a small sample of self-selected participants which may limit the generalizability of perceptions and experiences. Inclusion of large sample may have been provided [34]; (5) Although we included both compliant and non-compliant users, individuals who agreed to be interviewed may differ in their motivation or perceptions from those who declined participation [68]; This potential selection bias could have influenced the feedback on usability and acceptability; (6) Since the interviewer was part of the research team, participants may have provided socially desirable responses, which could have introduced response bias; (7) Although this study followed the mHealth Evidence Reporting and Assessment (mERA) checklist to assess feasibility, the absence of a complementary validated quantitative feasibility assessment tool (e.g., Feasibility of Intervention Measure) is acknowledged as a limitation; (8) Although we administered seven-day accelerometer-based physical activity measurements at baseline, 3 months, and 6 months [28], these measures did not fully capture self-reported compliance with the SmPh application prompts. Future trials should consider integrating accelerometer data with SMART-STEP application usage metrics to provide a more comprehensive understanding of user compliance among office workers.

## Conclusions

SMART-STEP application was developed at a low cost, that can be adapted in low-resource settings. The usability and acceptability decay after nine weeks of usage strongly emphasize that technology based behavioral intervention may be interesting & seem convenient in the short-term. However, to increase the adherence in the long term, the application-based prompts should be used in combination with counselling and environmental modification strategies.

## Supplementary Information

Supplementary Material 1.

## Data Availability

Data extraction sheet will be made available based on a reasonable request to the corresponding author.
